# Epileptic seizures caused by encephalomalasic cysts following radiotherapy: a case report

**DOI:** 10.4076/1757-1626-2-7026

**Published:** 2009-07-16

**Authors:** Fatih Serhat Erol, Bekir Akgun

**Affiliations:** Department of Neurosurgery, Firat UniversitesiTip Fakultesi, Firat Tip Merkezi, Beyin Cerrahi Klinigi, 23200, ElazigTurkey

## Abstract

**Introduction:**

Cerebral radionecrosis is a delayed and rarely observed complication of radiotherapy. Cerebral radionecrosis may occur as cystic encephalomalasic formations which cover the intracranial region. These cysts may, in rare cases, become quite large. They may cause drug-resistant seizures, neurological deficits and consciousness disorders.

**Case presentation:**

A 55-year-old, Turkish female patient was admitted to hospital with seizure, consciousness disorder and weakness in the right side of her body. The patient had history of an operation in the left maxillary area due to basal cell carcinoma 7 years previously and then history of radiotherapy due to relapse 2 years later the operation. The patient had large cystic encephalomalasic lesions. Despite steroid and dual antiepileptic treatments, the patient's complaints had significantly worsened and seizures continued. Surgical treatment resulted in a significant improvement.

**Conclusion:**

This report underlines the significance of surgery in cerebral radionecrosis treatment in well-selected cases using appropriate approaches.

## Introduction

Post radiotherapy cerebral radionecrosis development is a rare complication which occurs months or even years after treatment. This complication was associated with dose and fractions [1-3]. Various radiation-based grey and white matter changes may occur within the brain. Demyelinization and edema are common [2,4,5]. Therefore, the contribution of the effects of steroid treatments were observed in various radionecrosis cases [2,4]. However, cerebral encephalomalasic cyst development may worsen the neurological status of patients due to its significant mass effect. In patients who have such a significant mass effect, especially if the lesion is cystic, surgical approaches may provide significant contributions to the clinic of the patients [1,2,6]. We present the case of a radionecrosis patient in whom we obtained positive results with surgical treatment.

## Case presentation

The 55-year-old, Turkish female patient experienced generalized tonic clonic seizures which started 2 years previously and had become persistent during the last 3 months, despite antiepileptic treatment. In addition, the patient had complained of speech defects, forgetfulness, perception deficit for 2 months, cognitive disorders and a weakness in the right side of the body for the 2 days prior to admission. The patient had history of an operation in her left maxillary area due to basal cell carcinoma 7 years previously and then history of radiotherapy due to relapse 2 years later the operation. She had treated with a total dose of 56 Gy radiation, administered in 15 fractions to her left maxillary region, and she had no chemotherapy history.

**Figure 1.**
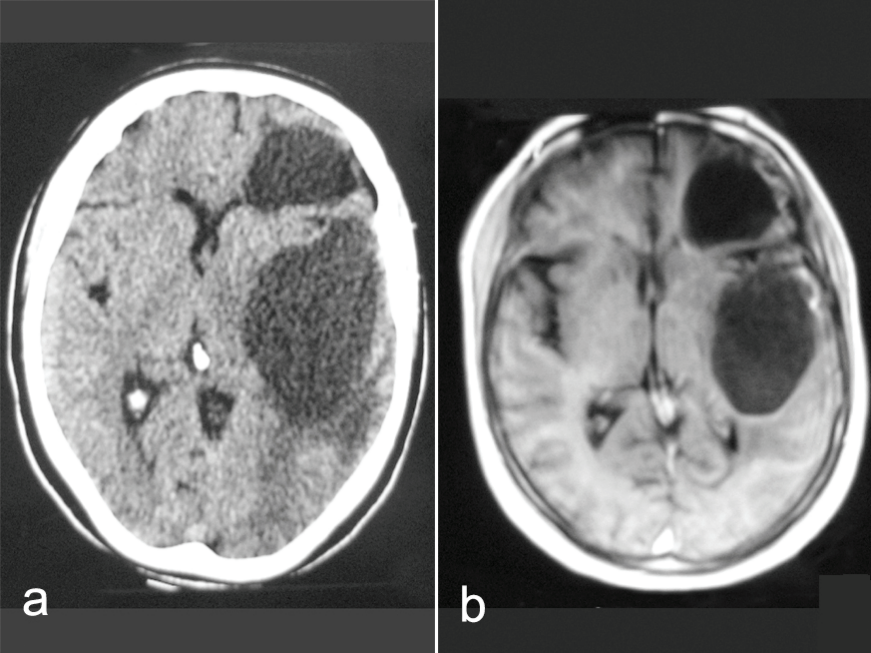
**(a)** Cranial CT scan showed hypodense lesions in the left frontal lobe and in the left temporal lobe. No significant edema was observed around the lesions, which compressed the left lateral ventricle and did not show contrast enhancement. **(b)** Cranial MR imaging showed in the left frontal region and in the left temporal region cystic lesions.

During neurological examination it was found that the patient experienced confusion and disorientation. However bilateral papilledema was detected on neurological examination. In addition, the patient had right hemiparesis. Her muscle strength grade was 3 (active movement against gravity) in the right side of her body. A cranial CT scan showed hypodense lesions in the left frontal lobe (3 × 4 cm) and in the left temporal lobe (6 × 4 cm). No significant edema was observed around the lesions, which compressed the left lateral ventricle and did not show contrast enhancement (Figure 1a). Cranial MR imaging showed in the left frontal region 3 × 4 cm and in the left temporal region 6 × 4 cm lesions, which were hypointense in T1 (Figure 1b), hyperintense in T2-weighted images. These lesions observed with mild peripheral contrasting. They made us think about cystic masses. An EEG showed reduced bioelectrical activity in the left frontal and left temporal regions. A course of antiepileptic treatment was organized for the patient. The patient, who was using 2 × 500 mg Sodium valproate, was given dual antiepileptic therapy, starting with 3 × 100 mg Phenytoin. In addition, steroid treatment was begun. However, despite this treatment, the patient experienced loss of consciousness, a generalized tonic-clonic seizure, an increase in right hemiparesis. Her muscle strength grade became 2 (active movement with gravity eliminated). As the result of these, the patient was taken to surgery. She underwent a left temporal craniotomy. Transcortical cystic fluid was reached. The content of the cyst was aspirated. In the left frontal region, craniectomy was performed to a certain degree and the cystic fluid was aspirated.

Under microscopic examination of the cyst fluid, blood elements, an amorphous substance consisting of fibrinoid and hyaline material, low number of histiocyte and lymphocyte cells were observed. In the examination of material taken from the walls of the cyst and surrounding brain parenchyma, perivascular lymphocyte infiltration and macrophages were observed and hemosiderin loaded macrophages were found. These findings made us think of reactive gliosis. Also there were no evidence of neoplastic cells in the examination of cyst fluid, cyst wall and surrounding brain parenchyma.

**Figure 2.**
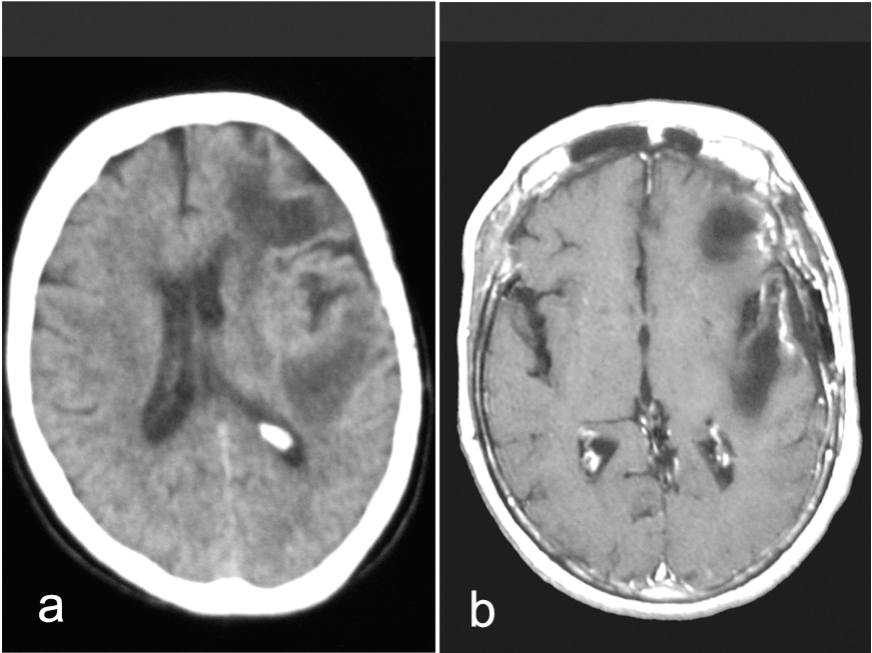
In postoperative CT **(a)** and MR **(b)** images were observed that both cystic masses got smaller.

In the early post-operative period, significant improvements were observed in the patient's consciousness, speech and right hemiparesis. Her muscle strength grade became 4 (active movement against gravity and resistance). The patient, whose follow-up still continues at our clinic, has not been experienced any postoperative seizure or neurologic deterioration. In postoperative follow-ups using images made for control purposes, it was observed that both cystic structures got smaller (Figure 2a and 2b).

## Discussion

Previous studies have reported that delayed cerebral radionecrosis has been observed after radiotherapy for various malignities such as nasopharyngeal cancers, brain tumors, maxillary carcinomas, ear carcinoma and following stereotactic treatment of tumors or vascular malformations [1-9].

The actual incidence of radiation necrosis in the brain is uncertain, but is approximately 5% [1,2]. Radiation necrosis can generally occur months, even years after radiotherapy [1-3]. Radionecrosis may occur more than 10 years after the initial radiotherapy. Because of the delayed fashion there is a belief that the actual incidence of late radionecrosis may be higher than the reported and expected ratios [1]. Accurate determination of the incidence of delayed radionecrosis would require long-term imaging follow-up.

According to their occurrence times, radiation encephalopathies are analyzed under 3 groups: 1. *Acute RE*, which develops a few days after radiation at the end of the radiotherapy; 2. *Sub-acute RE*, which occurs a few weeks to 6 months after the therapy; 3. *Delayed RE,* which takes place 6 months to possibly years after the therapy. Delayed RE is generally irreversible and sometimes even fatal. Temporal lobe damage is the most common and most serious condition in radiation encephalopathies. This constitutes 65% of the deaths caused by this medical status [3].

In recent studies radiation necrosis was demonstrated to vary depending on the total radiation dose, frequency, dose per fractions, duration of administration and the size of the exposed area [2,10]. A total dose of 20 Gy may be enough to cause radionecrosis. Various clinical studies suggested that higher total doses of radiation are associated with earlier formation of cerebral radionecrosis [1,10]. In addition, it was indicated that, patients who received a higher fractional dose had a significantly higher incidence of radionecrosis than those treated with a higher total dose but a lower fractional dose. For this reason, it was stressed that fractional dose is a very important factor affecting cerebral radionecrosis development [1,2,10]. Most authors agree that a total dose of 55 Gy, administered in fractions of 1.7 to 2 Gy is tolerated by the central nervous system. However, it should be remembered that the degree of radiosensitivity can show variations between individual cases [2]. Moreover, it is thought that, in addition to radiotherapy, neurotoxic chemotherapy agents (such as cisplatin or vincristin) increase the risk of this complication [2,7]. Our patient had treated with a total dose of 56 Gy radiation, administered in 15 fractions. However, our patient had no chemotherapy history.

The pathophysiology of cerebral radionecrosis is not well known. However, 3 main mechanisms were underlined. These are: 1.The occurrence of vascular lesions may be responsible for local thrombosis and cerebral ischemia 2. Radiation can have a direct toxic effect on glial cells; 3. An autoimmune reaction can occur which can cause damage in glial cells [2].

The symptoms and findings in our patient bear similarities to the principal symptoms and findings in previously reported cases. According to the literature, the possible symptoms and findings in cerebral radionecrosis patients are headache, seizure, vomiting, difficulty in maintaining concentration, loss of consciousness, dysphasia, walking disorders, confusion and hemiparesis [1,2,6-8]. Izawa *et al.* [8] reported that in their series in which cyst formations were developed, the most common presentation was headache and the second most common symptom was seizure. There were also examples in which patients had been asymptomatic. In the same series they reported that the patients who experienced deteriorations in their neurological status gained many benefits from surgical treatment. For asymptomatic patients, they suggested follow-up.

Since cerebral radionecrosis does not have many specific findings its diagnosis with imaging technologies presents some difficulties. It is difficult to distinguish it from a primary brain tumor, intracerebral relapse of a cutaneous tumor or from a cerebral tumor induced by radiation. MR spectroscopy and PET are advanced diagnosis methods recommended for the distinction of radionecrosis and tumor recurrences [1-3,5-7]. However, as the clinic of our patient had a rapid and progressive deterioration; we were not able to spare time for these advanced imaging methods. We applied our emergency surgery intervention with cranial CT and MR examinations which was carried out at the patient’s admittance. Cerebral CT examinations revealed that, in general, the areas of cerebral radionecrosis and, in particular, the cystic encephalomalasic lesions, had hypodensities. Cranial MR produced hypointense in T1 and hyperintense lesions in T2-weighted images. Peripheral contrasting can be observed not in CT but in cranial MR imaging. Late temporal lobe changes may include cerebral atrophy and micro cystic or macro cystic encephalomalacia. Cystic encephalomalacia may occur in the form of a large cyst or in the form of several small cysts [5]. Our patient’s CT and MR findings resembled to these knowledge.

In the reported cases, the histological analysis of the cyst wall include focal necrosis (containing fibrinoid or hyaline material) and astroglial proliferation without observing any tumor cells [1,7]. Also in the histological examination of our patient’s cyst content, there were findings which indicated a past focal necrosis. In the examination of the cyst wall and the surrounding brain parenchyma, reactive gliosis was determined. No neoplastic cells were detected in the cyst content, cyst wall or surrounding brain parenchyma. Along with radiotherapy history, this histological analysis was important for confirmation of our findings. Parallel to these findings, we were able to perform differential diagnoses of cerebral radionecrosis from intracerebral cystic tumor, malignancy induced by radiation and also intracranial metastasis of maxillary basal cell carcinoma in which the patient was previously diagnosed.

With Gammaknife surgery, radiation passes through the head along as many as 201 different trajectories, and, therefore, even distant areas of the brain are exposed to low doses of radiation. Because of the low doses of the radiation, in the relatively remote areas from the treatment area can’t be associated with delayed radionecrosis, but this can be associated with secondary tumor formation for the interval of 20 to 25 years [11]. Whereas development of encephalomalasic cysts can be observed after either radiotherapy or radiosurgery at the adjacent regions of the treatment areas. Wang PC *et al.* [6] reported in their patient, a left temporal lob encephalomalasic cyst which was developed after application of radiotherapy for left eksternal ear ca. Mineura K *et al.* [7] noted the development of bilateral frontal encephalomalasic cysts in a patient, after radiotherapy for an olfactory neuroblastoma. Pollock and Brown [12] reported their series with 6 patients, whom AVMs were treated with stereotactic radiosurgery, had delayed cyst formations at the adjacent areas of their AVMs.

Since it was known that free oxygen radicals have a role both in edema and necrosis development, the benefits of using antioxidants such as melatonin and vitamin E for protection purposes were indicated, especially in animal experiments [10]. However, familiar treatment approaches which can be clinically beneficial in radionecrosis cases consist of surgical treatment or corticosteroid treatment [1,2,10]. As vasogenic edema commonly accompanies radionecrosis cases, clinical cures and the patients who benefited from corticosteroid treatment were reported [2]. However, in patients with symptoms related to mass effect and especially in cystic radionecrosis cases, surgical treatment is generally recommended [1,2,6,8]. In this present case despite initiating steroid and antiepileptic treatments, the progressive deterioration in the patient’s clinic could not be prevented. For this reason, the patient was admitted to surgery and the aspiration of the existing cysts was carried out.

Wang *et al*. [6] reported that in a patient in whom a cystic encephalomalasic lesion developed after RT, following stereotactic aspiration of the cyst, they had successful results in external drainage with an Ommaya reservoir. Pollock and Brown [12] applied placement of stereotactic cystoperitoneal shunts or cyst excision for patients with recurrent cysts. According to these reports, in case of a relapse, encephalomalasic lesion development with occurring a deterioration in the clinic of our patient, it would be possible to try the cyst excision. Also employ the Ommaya reservoir technique or cystoperitoneal shunt after the cyst aspiration would be possible. Fortunately there was no experience of neurological deterioration or recurrent cyst enlargement in imagings’ during the follow-ups of our patient.

Patients treated with radiotherapy or radiosurgery, are at the risk for delayed cyst formation. Because of this, follow-up examinations and imagings are necessary years after the procedure. Patients with incidentally discovered micro cysts were followed up with serial imaging, whereas patients with symptoms related to mass effects from the significantly enlarged cysts underwent surgery intended to decompress the cyst. Cyst aspiration was performed initially, as the simplest operation possible for cyst treatment. In case of a relapse placement of Ommaya reservoir or cystoperitoneal shunt may provide permanent diversion of the cyst fluid. Also, if the structure and the location of the cyst is convenient, cyst excision may be preferred.

In epilepsy patients, quality of life decreases and mortality rate increases when compared to the general population. A positive correlation was indicated between the number of seizures and mortality. For this reason, the purpose of epilepsy treatment should be to provide the patient with a life without seizures [13]. It is known that surgical approaches have roles, especially in drug-resistant epilepsy. In cerebral radionecrosis cases, epilepsy can develop as a result of temporal lobe influences or encephalomalasic cyst formation. In patients with temporal lobe epilepsy, amygdalo-hippocampectomy operations have roles; in patients who have epilepsy depend on encephalomalasic cyst, surgical treatments for the removing of the cyst have roles [8,9].

## Conclusion

In conclusion, cerebral radionecrosis is a rare complication of radiotherapy which occurs months or even years after treatment. The isolated use of corticosteroids can affect a clinical cure in some cases. However, in cystic radionecrosis cases, surgical approaches such as cyst excision or aspiration should also be considered. Particularly in patients like ours, who experienced drug-resistant seizures, deterioration in consciousness, and deterioration in hemiparesis, if the structure and the location of the lesion is convenient, we suggest surgical intervention without delay.

## Abbreviations

CT: Computed tomography
MR: Magnetic resonance
PET: Positron emission tomography
RE: Radiation encephalopathies
